# Effect of Multiple Freezing/Thawing Cycles on the Structural and Functional Properties of Waxy Rice Starch

**DOI:** 10.1371/journal.pone.0127138

**Published:** 2015-05-27

**Authors:** Han Tao, Juan Yan, Jianwei Zhao, Yaoqi Tian, Zhengyu Jin, Xueming Xu

**Affiliations:** 1 School of Food Science and Technology, Jiangnan University, Wuxi, PR China; 2 State Key Laboratory of Food Science and Technology, Jiangnan University, Wuxi, PR China; 3 Synergetic Innovation Center of Food Safety and Nutrition, Jiangnan University, Wuxi, Jiangsu Province, China; National Taiwan University, TAIWAN

## Abstract

The structural and functional properties of non-gelatinized waxy rice starch were investigated after 1, 3, 7, and 10 freezing/thawing cycles. Freezing caused an increasing damaged starch from 1.36% in native waxy rice starch to 5.77% in 10 freezing/thawing-treated starch (FTS), as evidenced by the cracking surface on starch granules. More dry matter concentration was leached, which was characterized by high amylopectin concentration (4.34 mg/mL). The leaching was accompanied by a decrease in relative crystallinity from 35.19% in native starch to 31.34% in 10 FTS. Freezing treatment also led to significant deviations in the functional characteristics, for instance decreased gelatinization temperature range, enthalpy, and pasting viscosities. The resistant starch content of 10FTS significantly decreased from 58.9% to 19%, whereas the slowly digested starch content greatly increased from 23.8% in native starch to 50.3%. The increase in susceptibility to enzyme hydrolysis may be attributed to porous granular surface, amylopectin leaching, and the decrease in the relative crystallinity caused by freezing water.

## Introduction

Freezing technology is a suitable method to retard some bread making measures and to produce fresh bread available in the retail stores after baking, or to make frozen goods available that the consumer can bake at home while required. However, freezing procedure provoked various physical and chemical damages to the product [1]. Protein network and starch granules were mainly responsible for these changes in qualities [2]. There were many reports on the retrogradation properties of starch, a major constituent in bread, which took parts in the bread making process through assimilating water [3]. Extreme levels of damaged starch rose the water absorption ability of flours, forming problems throughout dough handling and fermentation [4]. Hence, flour with more than 7% damaged starch should not be used in bread making from frozen dough [5]. From the technological perspective, function of starch was the most significant in bread firming. Freezing resulted in a high degree of syneresis in gelatinized starches and then accelerates retrogradation that was characterized by the hard texture of frozen products [6,7]. Gelatinized system was a suitable approach to explore the changes in food products, except in frozen raw material. A series of physical phenomena and chemical reactions occurred during freezing and thawing processes, facilitating several irreversible structural and functional transformations during cooking and cooling periods [1]. A frozen dough (stored at −30°C and −40°C) showed an increase in the crystallinity extent of dough starch, which was affiliated to starch retrogradation [8]. To date, information on the relationship between crystalline structures and thermal and in vitro starch nutritious fractions [rapidly digested starch (RDS), slowly digested starch (SDS), and resistant starch (RS)] in starch was rare. Szymonska et al. [9] reported that deep freezing/thawing (F/T) treatment altered water distribution in potato starch granules and increased their surface coarseness.

Gluten-free breads were instantly accessible in the market because of celiac disease and other allergic reactions/intolerances through gluten consumption. These breads were mainly formed by rice flour and hydrocolloids instead of gluten network [10]. Several studies considered the rheological and textural characteristics of gluten-free dough following storage in sub-zero conditions. However, few analyzed the structural and functional properties of individual components, such as rice starch. Mezaize et al. [11] reported that the firmness of frozen—thawed gluten-free dough was greater than that of fresh dough. They also indicated that gluten-free breads achieved by frozen dough had lower specific volumes and harder crumbs than those of ordinary gluten-free breads. Leray et al. [12] also reported that the gumminess, cohesiveness, and springiness of frozen gluten-free dough increased with storage time, whereas the storage modulus and loss modulus decreased. Dough formulations included various components that had complicated changes during the bread making process, as well as during bread storage, which made an extremely complex phenomenon.

Waxy rice starch, which contained only amylopectin (AP), served as a simple model to determine the role of starch in deteriorating frozen products. Gelatinization properties, pasting behaviors, and in vitro digestibility of waxy rice starch were affiliated to amylopectin structure. A non-gelatinized system method was employed to elucidate the effects of freezing on the properties of waxy rice starch before cooking to control a better production process.

## Materials and Methods

### Materials

Commercial waxy rice starch was provided by Suzhou Youi Foods Co., Ltd (Jiangsu, P. R. China). Moisture contents were determined based on weight loss at 105°C to constant weight of 1.0 g of accurately weighed samples [13]. All solvents were analytical grade and provided by Sinopharm Chemical Reagent Co., Ltd. (Suzhou, China).

### Freezing/thawing treatment

About 30 g of waxy rice starch in excess water (45 mL) was stored at −20°C for 24h after pre-freezing at −30°C in a refrigerator to obtain an inner core temperature of −18°C. The sample was then thawed to equilibrate for 2h at an ambient temperature (25°C). This F/T cycle was repeated for different cycles (1, 3, 7, and 10, respectively) before removing the supernatants by centrifugation at 2200 × g for 20 min. The starch pellets were dried at 40°C for 2 days and passed through a 100 mesh sieve for the following analysis. The collected supernatant solutions after centrifugation were used for composition analyses.

### Scanning electron microscopy (SEM)

Freeze-dried starch pellets were mounted on a tray and coated with gold to detect micrographs. The morphology of native and F/T samples was observed under a Hitachi S-4800 field emission scanning electron microscope (Hitachi, Japan). The samples were viewed at an accelerating voltage of 10 kV, and representative micrographs from all the samples were selected for illustration.

### Damaged starch content

The damaged starch content (%) were assessed by an fungal enzymatic standard method [14] and the enzyme from *Aspergillusoryzae* (Sigma Chemical Co., USA) was applied.

### Dry matter concentration

The dry matter concentration of the starch supernatant was estimated using a vacuum oven keeping at 105°C for one night until their dry weights were recorded. Then it was measured using the following equation

$$Drymatterconcentration(\%)=\frac{drymatterweight(\text{g})}{\mathrm{supernantant}(\text{g})}\times 100\%$$ (1)

### Analysis of dual-wavelength spectrophotometry

About 10 mg of freeze—dried supernatant obtained by Labconco FreeZone (Labconco, USA) was dissolved in a 1 mL of 0.1 mL of ethanol and 0.9 mL of 1 M NaOH aqueous solution. Then the amylopectin concentration was determined by dual-wavelength spectrophotometry with iodine as the coloring agent [15]. The optical absorbances of I_2_-amylopectin were 551 and 761 nm, as determined using a spectrophotometer (UV1100; Beijing Ruili Instrument Company, Beijing, China). The distilled water was selected as reference. The amylopectin concentration was calculated using the following formula. All analyses were performed in triplicate.

$$W=\frac{\left(OD_{551nm}-OD_{761nm}\right)-0.0127}{0.0134\times M\times \left(1-X\right)\times 10}$$ (2)

Where W is amylopectin concentration (mg/mL), M is the weight of the sample powder (g), X is the water content of the sample powder (g/g), and 10 is the coefficient for the uniform unit.

The other coefficients were acquired from the regressive equation of amylopectin.

### X-ray diffraction (XRD)

The XRD patterns of starch powders were recorded in a scanning range of 4° to 40° at room temperature. A Bruker D8-Advance XRD instrument (Bruker AXS Inc., Germany) was run in a scanning speed of 4°/min with radiation at a set voltage of 40 KV and current of 30 mA, respectively.

### Differential scanning calorimetry (DSC)

Thermal properties were determined by a SIINT instrument (X-DSC 7000 model; Japan). Prepared samples (3mg, db.) were sealed into aluminum pans in blends of 6 μL of distilled water and equilibrated at 4°C for 24h. An empty pan was used as a reference; the sample pans were heated at a range of 20°C to 90°C with a constant rate of 10°C/min using nitrogen gas at a flow rate of 80 mL/min. The onset (T_o_), peak (T_p_), conclusion temperature (T_c_), and enthalpy (ΔH) of gelatinization were obtained by TA Rheology System Software Muse, version 1.6 (SIINT, Japan, 2012). Each sample was run in triplicate.

### Viscoamylograph profiles of starch

Pasting curves were powerful tools to depict starch functional properties. A Brabenderviscograph-E (Brabender GmbH & Co. KG, Germany) was used to measure starch viscosity. An aqueous dispersion of starch suspension (7%, db.) was prepared in the viscosity measure cup. For the mixture with distilled water, the slurries were held at 30°C for 1 min, heated at a rate of 3.0°C/min to 95°C, maintained at that temperature for 30 min, cooled to 50°C at a rate of 3.0°C/min, and held at 50°C for 30 min. The average values for peak viscosity (PV) (BU), trough viscosity (TV) (BU), final viscosity (FV) (BU), pasting temperature (PT) (°C), breakdown (BV = PV-TV) (BU), and setback viscosity (SV = FV-TV) (BU) were obtained for each sample from triplicate Brabender viscosity measurements.

### In vitro starch digestibility

Starch digestibility was determined by the procedure described by Englyst et al with modifications. Porcine pancreatic α-amylase (0.24g, Megazyme) was dispersed in 0.1 M sodium acetate buffer (pH 5.2) and centrifuged at 1500 g for 10 min. Then 0.1 mL of amyloglucosidase (3260 units/mL, Megazyme) was added to the supernatant (8 mL). Starch (200 mg) and 4 mL of 0.1 M sodium acetate buffer (pH 5.2) were added to each test tube, followed by the prepared enzyme solution (1 mL). The mixture was incubated in a shaking water bath (37°C, 180 rpm). Aliquots (0.1 mL) were taken at 20 min and 120 min and mixed with 0.9 mL of 95% ethanol. The hydrolyzed glucose content was measured by the 3, 5-dintrosalicylic acid (1%, w/v) method. There were three starch fractions classified by the rate of hydrolysis: RDS (digested within 20 min), SDS (digested between 20 and 120 min), and RS (undigested starch after 120 min).

### Statistical Analysis

All experiments were subjected to an analysis of variance (ANOVA) by Duncan’s test (p < 0.05) was conducted using the SPSS 16.0 software (SPSS, Inc., Chicago, IL, USA).

## Results and Discussion

### Starch granule morphology and component analyses

**Fig 1:**
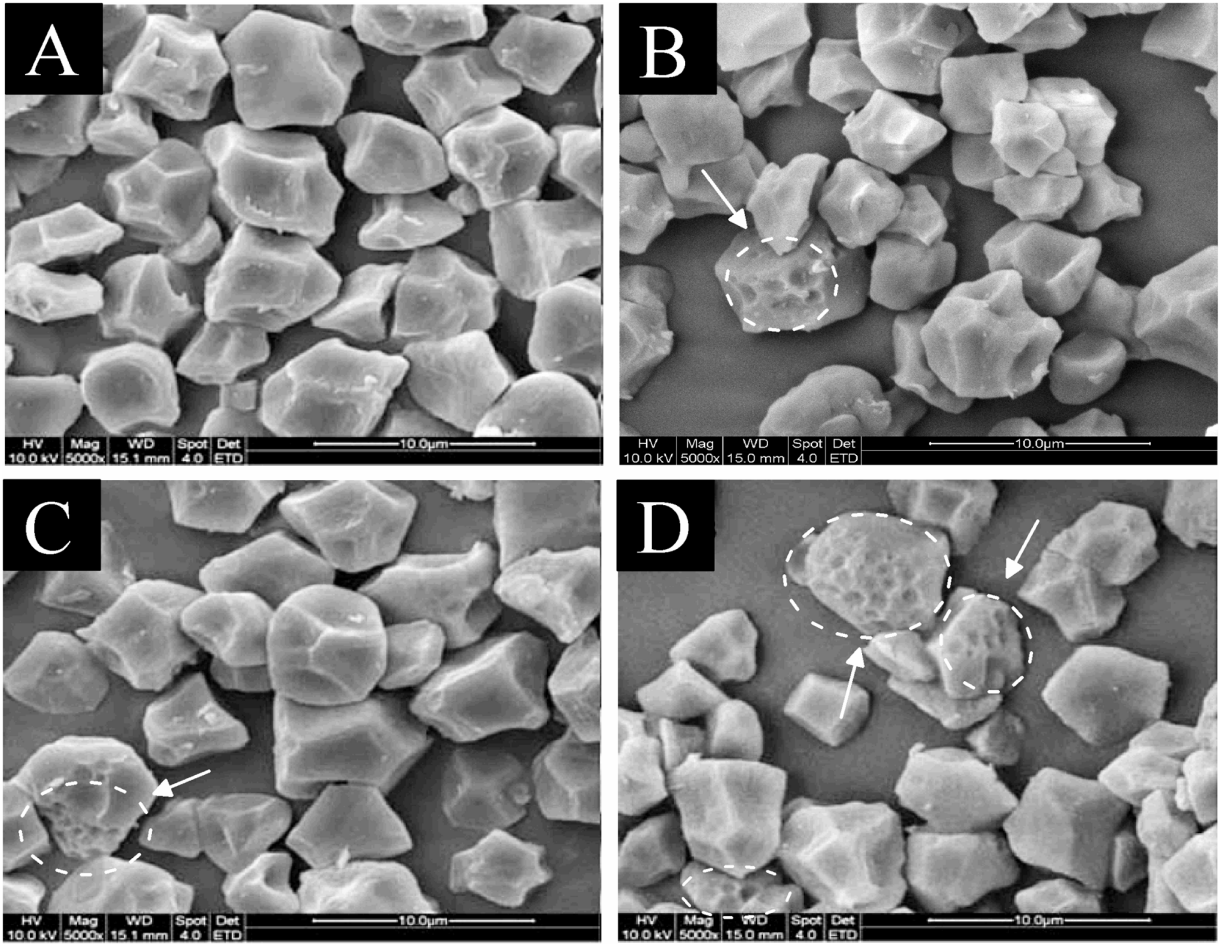
SEM micrographs of (A) native waxy rice starch (NWS), (B) 3 freezing/thawing-treated starch (3 FTS), (C) 7 freezing/thawing-treated starch (7 FTS), and (D) 10 freezing/thawing-treated starch (10 FTS).

Fig 1A–1D illustrated the SEM micrographs of native and F/T-treated waxy rice starch (FTS). Native waxy rice starch (NWS) displayed a small (2 μm–7 μm) angular polyhedral shape and a smooth surface (Fig 1A). After 1 F/T cycle, some pores were observed on the granule surface (Fig 1B). These changes became more evident after multiple exposures to ice crystals during freezing (Fig 1C–1D), which could be interpreted as followed. During the freezing process, internal or external freezing water applied a high pressure to starch granules because of phase transformation. This pressure enabled the granules to compress and crumble by ice matrix, resulting in the appearance of hollows on the granule surface [9]. In our case, waxy rice starch did not aggregate after freezing because its small granule size exhibited more resistance to compression.

The pitted appearances were consistent with the altered damaged starch content, which was greatly increased from to 1.36% in NWS to 4.57% in 10 FTS (Table 1). The dry matter content of the supernatants significantly increased from 0.83% in NWS to 5.17% in 1 FTS because of the damage and prompted at 8.83% during F/T cycles up to 10 (Table 1). An increasing tendency was also observed on the amylopectin concentration in supernatants, which increased from 0.58 mg/mL to 4.34 mg/mL. The F/T process continued to act on the granules over time and gradually disrupted the surface, facilitating damage on the granules and leaching materials. Similarly, Barrera et al. [16] reported that leached material increased with damaged starch content and became proportionally rich in amylopectin. Physical damage promoted hydration and swelling of starch granules; hence, a large number of starch granules could spontaneously gelatinize. Thus, the centrifuged supernatants were rich in some material that partially diffused out of damaged granules.

**Table 1:** Summary of results from DMC, AP, DS and RC determination for native and freezing/thawing-treated starches ^a^.

Samples	DMC (%)	AP (mg/mL)	DS (%)	RC (%)
NWS	2.83± 0.53c	0.58 ± 0.17c	1.36 ± 0.11c	35.20 ± 0.31a
1 FTS	5.17 ± 0.32b	2.46 ± 0.16b	3.51 ± 0.10b	33.92 ± 0.29ab
3 FTS	7.01 ± 0.21a	3.63 ± 0.12b	4.19 ± 0.13ab	32.00 ± 0.25b
7 FTS	7.46 ± 0.56a	3.83 ± 0.12b	4.50 ± 0.13a	31.34 ± 0.28bc
10 FTS	8.83 ± 0.54a	4.34 ± 0.13a	4.57 ± 0.15a	31.30 ± 0.25bc

All data represent the mean of three determinations.

Values are means ± standard derivation.

Values with the same letters in the same column are not significantly differently (P < 0.05).

^a^ DMC, AP, DS, and RC are the dry matter concentration, amylopectin, damaged starch, and relative crystallinity, respectively.

The DMC and AP contents estimate in supernatants.

### Crystalline structure

**Fig 2:**
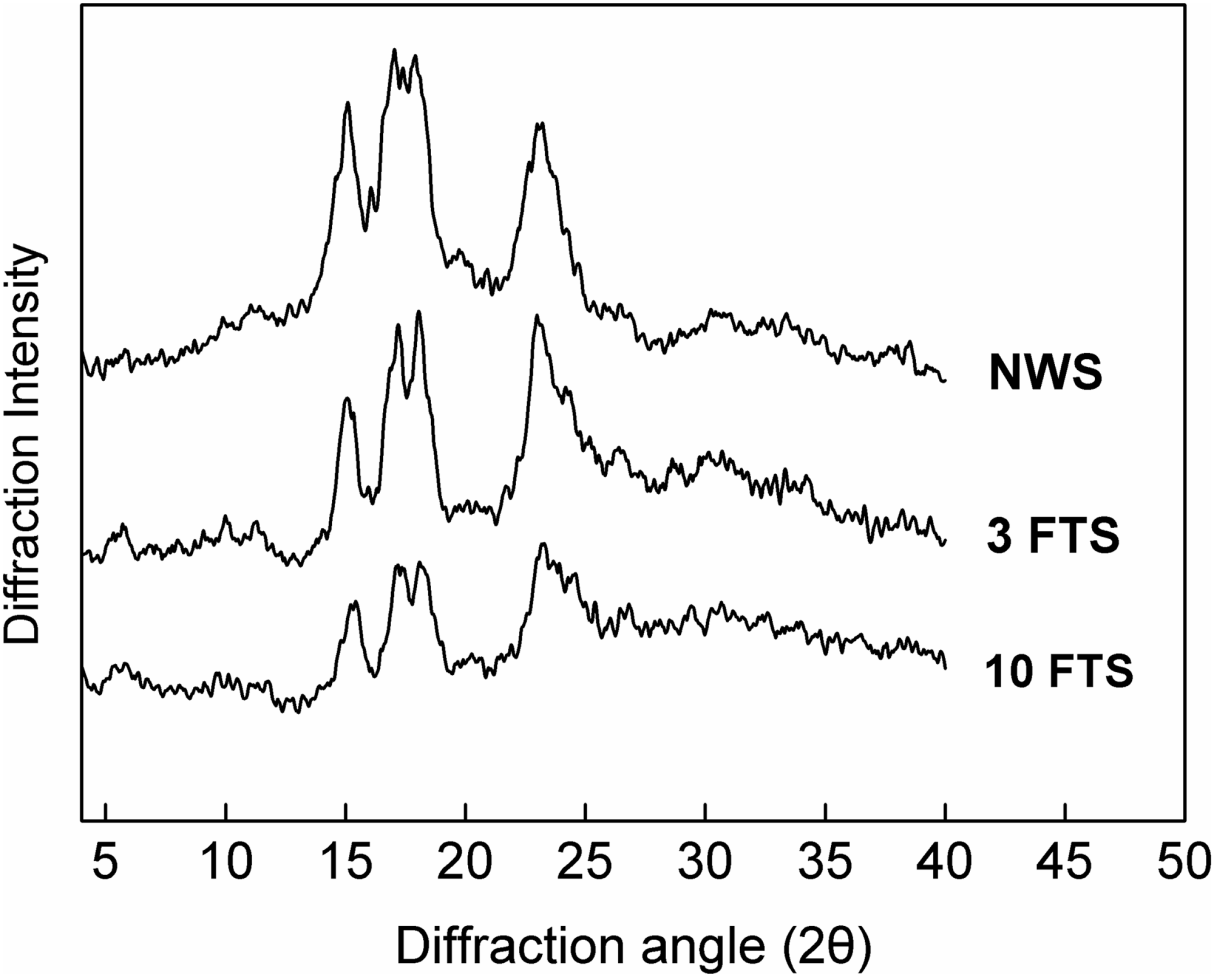
XRD patterns of native and freezing/thawing-treated waxy rice starches.

The XRD diffractograms were analyzed using Jade 5.0 software (Materials Data Inc., Livermore, CA, USA), and the relative crystallinity was calculated by dividing the area of the peaks by the total area of the diffractogram [17]. No significant changes were observed on the crystallinity profiles which were the typical A-type pattern of cereal starches (Fig 2). The relative crystallinity decreased from 35.2% in NWS to 31.3% in FTS after 10 cycles (Table 1) because of freezing. This decreased relative crystallinity could be attributed to factors, such as damaged starch, disruption of starch granules, and amylopectin leaching induced by freezing water. Li et al. [15] reported that the degree of starch crystallinity generally was in positive relation with the damaged starch granules. The crystalline structure of starch could be completely destroyed by prolonged grinding, as confirmed by the absence of defined peaks in an X-ray diffractogram. Szymonska and Wodnicka [18] also reported that damage was usually accompanied by the loss of the double helices in the amylopectins, which affected the organization of starch crystallites. Amylopectin was generally considered responsible for starch crystallinity [19]. Hence, the relative crystallinity in waxy rice starches deviated with amylopectin content and the level of starch damage.

### Thermal properties

Table 2 summarized the transition temperatures T_o_, T_p_, and T_c_, and the ΔH. The T_o_, T_p_, and T_c_ of the NWS were 72°C, 77°C, and 85°C, respectively. Freezing treatment (1 F/T) decreased the T_c_, but did not greatly affect the T_o_ and T_p_. The gelatinization temperature range (T_c_–T_o_) and ΔH significantly decreased from 12.76°C to 9.22°C, and from 15.43 J/g to 6.01 J/g, respectively, at the first F/T cycle. Slight changes were observed in T_o_, T_p_, and T_c_, and ΔH between freezing/thawing-treated starches (FTS).

**Table 2:** Gelatinization thermodynamic parameters of native and freezing/thawing-treated waxy rice starches ^a^.

Starch	T_o_ (°C)	T_p_ (°C)	T_c_ (°C)	T_c-_T_o_ (°C)	ΔH (J/g)
NWS	72.57 ± 0.25a	77.04 ± 0.53a	85.33 ± 0.38a	12.76 ± 0.67a	15.43 ± 0.40a
1 FTS	72.07 ± 0.42a	76.38 ± 0.26a	81.29 ± 0.23b	9.22 ± 0.38b	6.01 ± 0.37b
3 FTS	71.87 ± 0.53a	76.28 ± 0.24a	80.88 ± 0.52b	9.01 ± 0.33b	6.27 ± 0.54b
7 FTS	71.73 ± 0.36a	75.99 ± 0.33a	80.63 ± 0.50b	8.90 ± 0.43b	5.94 ± 0.42b
10 FTS	72.24 ± 0.42a	76.43 ± 0.36a	80.90 ± 0.61b	8.66 ± 0.51b	5.88 ± 0.52b

All data represent the mean of three determinations.

Values are means ± standard derivation.

Values with the same letters in the same column are not significantly differently (P < 0.05).

^a^ *T*_*o*_, *T*_*p*_ and *T*_*c*_ are the temperatures of the onset, peak and conclusion of gelatinization, respectively. *T*_*c*_-*T*_*o*_ is the temperatures range of gelatinization; and Δ*H* is the enthalpy change of gelatinization.

The differences in T_c_-T_o_ mirrored variations in the crystalline shape, size, degree of crystal perfection and the kind of starch chain intertwining that develop the double helical chains of starch crystallite [20]. Therefore, crystallinites could originate from the intertwining of the outer chains of amylopectin in amylopectin-rich starch. These crystallinites associated to form the ordered regions or ‘crystalline lamellae’ [21]. Compared with NWS, the narrow temperature range of starch after multiple F/T treatments may be attributed to amylopectin loss and may further decrease the imperfection of crystallinity. Starches with a low proportion of amylopectin can form a less perfect crystalline structure as evidenced by the gelatinization temperature [22]. Gidley and Bulpin [23] suggested that low gelatination temperatures corresponded to a decline on the stability of double helix. A similar experiment was conducted by Tester and Morrison [24], who compared the gelatinization properties of normal and waxy starches from barley and maize. They found that the gelatinization process of normal starches was completed earlier than that of waxy starches.

Waxy starches have larger gelatinization enthalpy than normal starches because starch with greater amylopectin amount had increased crystalline and less amorphous regions [25]. The enthalpy decreased with increasing damaged starch content because of multiple F/T cycles. Barrera et al. [16] reported that damaged starch hydrates spontaneously in cold water; therefore, particularly the native granules and fragmented granule fraction contributed to enthalpy. Decreased enthalpy could also be associated to decreasing relative crystallinity, which had a positive correlation with ΔH [26].

### Viscoamylograph profiles

**Fig 3:**
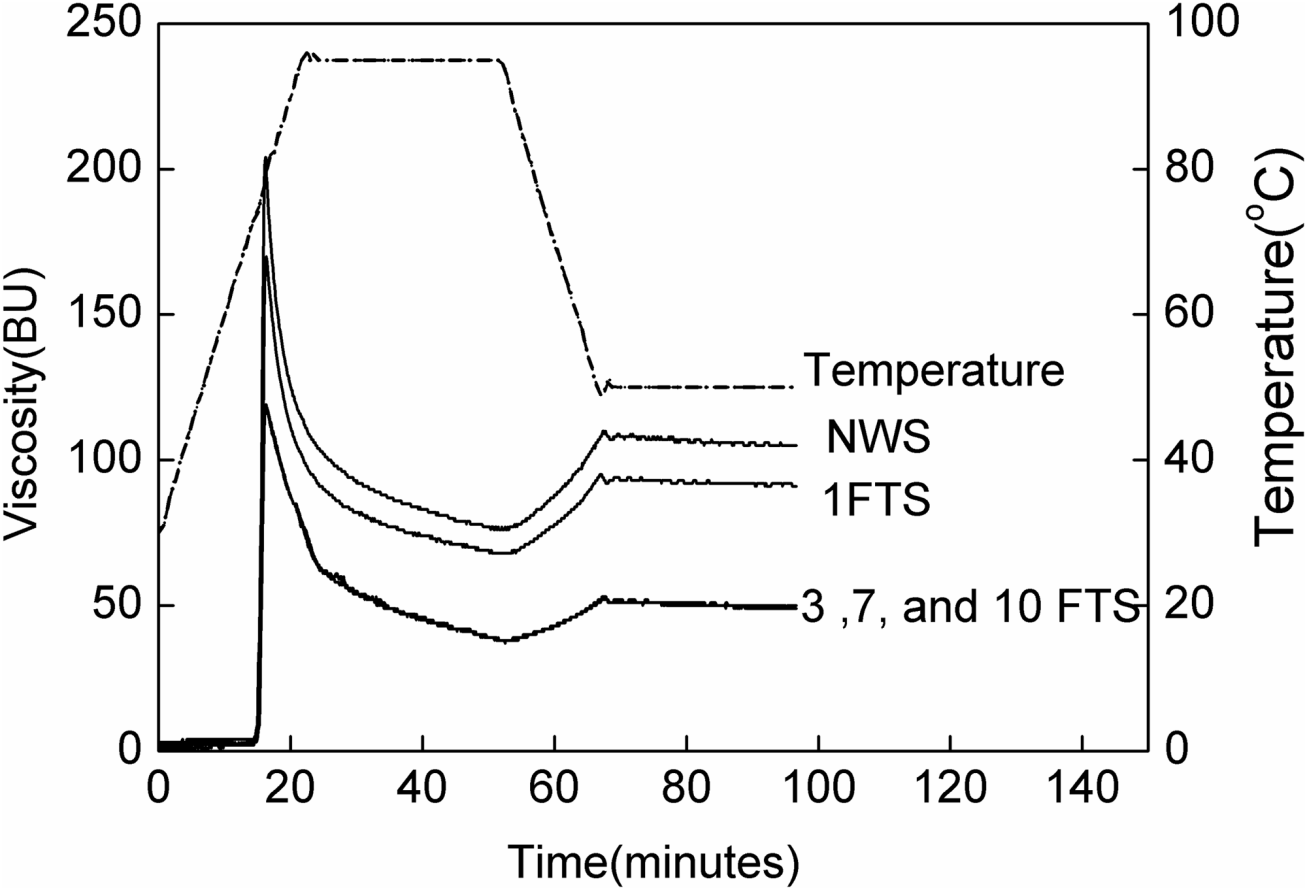
Pasting profiles of native and freezing/thawing-treated waxy rice starches.

The pasting outlines and characteristics of native and FTS were summarized in Fig 3 and Table 3, respectively. Freezing treatment facilitated a significant decrease in PV, BV, and FV, whereas no changes were observed in SV and PT. The increment of F/T cycles produced a 42.1%, 37.5%, and 58% reduction of PV, BV, and SV, respectively (Table 3). No significant changes were perceived in PT.

**Table 3:** Pasting characteristics of native and freezing/thawing-treated waxy rice starches ^a^.

Samples	PV (BU)	TV (BU)	BV (BU)	FV (BU)	SV (BU)	PT (°C)
NWS	204±17a	76±7a	128±16a	107±11a	31±5a	79.6±0.6a
1 FTS	169±11b	68±5ab	101±4ab	92±11ab	24±5ab	79.6±0.3a
3 FTS	118±14c	38±4a	80±6b	51±30b	13±7b	79.9±0.4a
7 FTS	119±12c	37±7a	82±5b	50±19b	13±8b	79.4±0.5a
10 FTS	118±11c	38±5b	80±5b	51±42b	13±4b	79.9±0.4a

All data represent the mean of three determinations.

Values are means ± standard derivation.

Means with the same letters in a column do not differ significantly (p < 0.05).

^a^ PV, TV, BV, FV, and SV are the peak viscosity, trough viscosity, breakdown viscosity, final viscosity, and setback viscosity, respectively. PT is pasting temperature.

Waxy rice starch mainly consisted of amylopectin without amylose—lipid complexes. Thus, pasting properties were influenced by amylopectin content and the integrity of the swollen starch granules. Because starch swelling was mainly property of amylopectin [24], high amylopectin was related with high peak viscosity [16,27]. The loss of amylopectin weakened the ability to form structure into the starch pastes, which agreed well with our present study. The reductions on breakdown viscosities indicated that freezing/thawing-treated starch developed viscosity but could not maintain the stability of paste viscosity. This difference would reflect amylopectin content.

The gel viscosity at the end of the test depended on the leached amylose concentration, occupied volume by the swollen granules, stiffness of the dispersed granules, and the attractive forces between granules and continuous phase [16]. The final viscosity was substantially low when no or less amylose was present in waxy rice starch, whereas a reduction in cold paste viscosity values was observed in frozen starches after repeated cycles. The final viscosity could be modified as a consequence of more damaged starch, which indicated a smaller occupied volume fraction of the dispersion.

### In vitro starch digestibility

The amount of waxy rice RDS, SDS, and RS nutritional fraction was presented in Table 4. Freezing/thawing treatment resulted in higher RDS and SDS contents and lower RS content compared to native waxy rice starch. RDS and SDS contents ranged from 17.3 to 23.8% in of the NWS and 37.6 to 50.3% in 10 FTS respectively. Meanwhile, the RS content was significantly decreased from 58.9% in NWS to 19.0% in 10 FTS.

**Table 4:** Proportions of RDS, SDS, and RS in samples ^a^.

Samples	RDS (%)	SDS (%)	RS (%)
NWS	17.3±0.8c	23.8±1.6d	58.9±1.6a
1 FTS	25.6±1.6b	38.5±0.7c	35.9±1.2b
3 FTS	31.7±1.7ab	43.1±0.9b	25.2±1.0c
7 FTS	35.0±1.1a	47.4±1.5ab	17.6±1.6d
10 FTS	37.6±0.4a	50.3±0.7a	19.0±1.3d

All data represent the mean of three determinations.

Values are means ± standard derivation.

Means with the same letters in a column do not differ significantly (p < 0.05).

^a^ RDS, SDS, and RS are rapidly digested starch, slowly digested starch, and resistant starch, respectively.

In vitro digestibility of starch comprised of enzyme diffusion into the substrate, enzyme adsorption to the substrate, and hydrolytic event [28,29]. This process was influenced by amylose to amylopectin ratio, crystalline structure, granule size and relative surface area, granule integrity, granule porosity, and structural inhomogeneities [30–32]. Compared with the intact native waxy starch granules with smooth surface structure (lacking of pinholes), frozen starch had a lower resistance to enzyme hydrolysis since a large relative surface area of damaged starch increased the exposure of the inner part of the starch granules; the inner part was normally more susceptible to enzyme hydrolysis than the granule surface [33]. A fast enzyme binding could exist in frozen starch, as evidenced by the low DSC enthalpy [15,34]. The low RDS content of waxy rice starches was also related to crystalline structure [32]. Chung et al. [30] reported that crystallites imposed a physical limit on enzyme accessibility. Thus, the freezing treatment greatly increased the RDS and SDS content, but decreased RS which could be attributed to several factors: great rigidity, extent of crystalline regions and cracking granular surface.

### Conclusions

This study showed that freezing/thawing (F/T) treatment had a substantial effect on the structural and functional characteristics of waxy rice starches. When waxy rice starch granule suspensions were frozen, a pressure chamber was developed because the internal or external ice crystals occupied more space than an equal amount of water. This pressure affected the structural and functional properties of waxy rice starch after multiple F/T cycles, causing the increased damaged appearance in the starch granules and amylopectin leaching of waxy rice starches. Moreover, freezing treatment prevented the increase in the relative crystallinity of starch. These substantial changes were accompanied by a decrease in gelatinized temperature range, enthalpy, and pasting viscosities with the F/T cycles. The removal of some materials may weaken the overall stability of starch granules, thereby leading to the significant changes to in vitro digestibility of freezing/thawing-treated starches. These experimental results provide useful information about the deteriorated qualities of frozen rice food.
